# EBI Search: providing discovery tools for biological metadata in 2025

**DOI:** 10.1093/nar/gkaf359

**Published:** 2025-05-05

**Authors:** Matthew Pearce, Prasad Basutkar, Renato Caminha Juaçaba Neto, Vijay Venkatesh Subramoniam, Kelsey Neis, Iva Tutis, Henning Hermjakob

**Affiliations:** European Molecular Biology Laboratory, European Bioinformatics Institute (EMBL-EBI), Wellcome Trust Genome Campus, Hinxton, Cambridge CB10 1SD, United Kingdom; European Molecular Biology Laboratory, European Bioinformatics Institute (EMBL-EBI), Wellcome Trust Genome Campus, Hinxton, Cambridge CB10 1SD, United Kingdom; European Molecular Biology Laboratory, European Bioinformatics Institute (EMBL-EBI), Wellcome Trust Genome Campus, Hinxton, Cambridge CB10 1SD, United Kingdom; European Molecular Biology Laboratory, European Bioinformatics Institute (EMBL-EBI), Wellcome Trust Genome Campus, Hinxton, Cambridge CB10 1SD, United Kingdom; European Molecular Biology Laboratory, European Bioinformatics Institute (EMBL-EBI), Wellcome Trust Genome Campus, Hinxton, Cambridge CB10 1SD, United Kingdom; European Molecular Biology Laboratory, European Bioinformatics Institute (EMBL-EBI), Wellcome Trust Genome Campus, Hinxton, Cambridge CB10 1SD, United Kingdom; European Molecular Biology Laboratory, European Bioinformatics Institute (EMBL-EBI), Wellcome Trust Genome Campus, Hinxton, Cambridge CB10 1SD, United Kingdom

## Abstract

The data resources provided by the European Bioinformatics Institute (EMBL-EBI) cover major areas of biological and biomedical research, giving free and open access to users ranging from expert to casual level. The EBI Search engine provides a unified metadata search engine across these resources. It provides a full-text search engine across over 6.5 billion data items, accessed through a user-friendly website and an OpenAPI-compliant programmatic interface. Here, we discuss recent developments and improvements in the service.

## Graphical Abstract

**Graphical Abstract:**
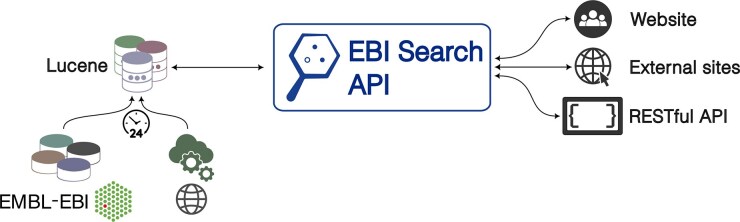

## Introduction

In 2024, the EBI Search engine received 2.3 billion requests from nearly 3 million unique hosts. Queries originated from the EBI Search web application and a number of other EMBL-EBI services, including ENA [1], RNAcentral [2], OmicsDI.org [3], Ensembl Genomes [4], and the COVID-19 Data Portal [5], searching over nearly 2 TB of indexed data representing 6.5 billion data items. The full dataset is indexed using a highly parallelized indexing pipeline running on a high-performance computing platform, with updates retrieved and processed on a daily basis.

Development of EBI Search has been continuous since its inception in 2006, working with teams across EMBL-EBI to improve and implement desired functionality and expand the framework. Over the last 19 years, it has provided free text search and cross-referencing capabilities across datasets from both within and outside EMBL-EBI, as well as enabling links to bioinformatics applications through the Job Dispatcher tools [6]. This paper discusses the latest updates and developments within the EBI Search engine.

## EBI Search

The EBI Search engine [7] uses Lucene Core (https://lucene.apache.org/core/) to index data spread across over 170 datasets (domains), and provides both a front-end and REST API to retrieve data from across those domains. Result sets may be retrieved with facet information, both simple and hierarchical, allowing the user to quickly identify the relevant results by filtering by date, organism, data type, etc. Data are stored with cross-references, which can be retrieved on an entry-by-entry basis, showing which additional datasets relate to a particular entry. Cross-references are bidirectional, meaning that they only need to be defined from one entry to be found when searching both referrer and referee.

Relevance scoring is an important part of data discovery, indicating how well a document matches the user’s search criteria. EBI Search has expanded its query options to enable filter queries through the API, allowing query clauses that are not included in the relevance score calculations. This results in faster execution and better relevance overall.

Bulk querying, allowing access to result sets larger than 1 million entries, has been expanded. It is now possible to stream results in tabular formats, enabling an entire result set to be downloaded using a single HTTP request. This facility is available on a subset of domains where it has been requested.

Improvements have also been made to the indexing pipeline. The parallelization strategy accounts for the memory and number of processors available, ensuring that the hardware is used as efficiently as possible. Changes to the workload managers in use at EMBL-EBI have also necessitated changes as the pipeline was re-worked to take advantage of new functionality while making best use of the infrastructure.

### User experience updates

The EBI Search website is an important entry point to the search engine, providing an accessible presentation of search results, as well as documentation and information about the EBI Search engine’s purpose within the wider EMBL-EBI organization.

**Figure 1.**
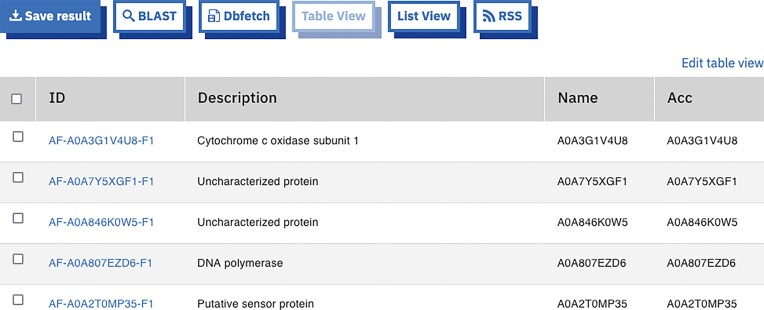
Table view of search results for query ‘domain_source:alphafold’, with controls (https://www.ebi.ac.uk/ebisearch/search?db=alphafold&query=domain_source:alphafold).

A new result visualization method has been implemented in EBI Search, enabling search results from individual domains to be displayed in a tabular format (see Fig. 1). This enhancement allows users to view all available column data associated with a domain within a structured table view. By default, the table initially presents five relevant columns, while users retain the flexibility to customize their view by selecting or deselecting additional columns as needed. Furthermore, all functionalities available in the list view—such as faceted searching, ORCID (Open Researcher and Contributor ID) claiming, and pagination—are fully supported in the table view. The column data for each domain are taken dynamically via the EBI Search REST API, ensuring that all relevant data for the selected page are retrieved and displayed in accordance with user preferences.

The data coverage presentation has been improved, using the FoamTree library (https://carrotsearch.com/foamtree/) to display the domain hierarchy both by number of entries (see Supplementary Fig. S1A and B) and by number of requests. This provides a colourful and interactive way of exploring the content covered. Individual blocks are sized logarithmically, so the largest categories do not dominate the display and make smaller categories difficult to see.

Request logging is an important part of understanding the usage and performance of the EBI Search platform over time. We have improved the logging process to allow us to evaluate the ranking of returned results, collating search terms with the entries users found most useful, which allows us to analyse and improve relevance. Logs are anonymized and no user identifiable data are recorded to preserve user privacy.

Documentation has been updated, and the link for the API documentation has been made more prominent on the base documentation page. The documentation for ORCID claiming has been re-written to improve clarity and is linked from the main page of the application. The Summary (S4) service API documentation is now included in the main API documentation page.

### Portal site developments

Though originally developed to index EMBL-EBI metadata, EBI Search can easily index and reference data from resources outside EMBL-EBI, based on a simple metadata format (http://www.ebi.ac.uk/ebisearch/XML4dbDumps.xsd). This makes it a valuable tool for the development of portal sites providing integrated discovery of data from within and beyond EMBL-EBI.

The Pathogens Portal [8] is one example of an online platform that uses this flexibility to integrate external data. It was designed to facilitate access to an extensive collection of biomolecular data related to pathogens. It serves as a critical resource for researchers by providing comprehensive data on various pathogen species and strains.

The core functionality of the Pathogens Portal is powered by the EBI Search API, which is utilized to aggregate and present data in a structured tabular format. The Pathogens Portal supports the ORCID claiming mechanism via EBI Search (see below), allowing researchers to link their work to their ORCID profiles. The platform includes data from other National Data Portals, which are stored and managed in EBI Search.

A template portal (https://ebi-wp.gitdocs.ebi.ac.uk/portal-starter-full/) has been developed to streamline the process of creating EBI Search-powered portals with a consistent look and feel and a set of common features including a fully functioning search system. It was originally derived from the COVID-19 Data Portal and has since been used as the basis for the Pathogens Portal and the EarlyCause Portal (https://portal.earlycause.eu/). There are now publicly available libraries on NPM (https://www.npmjs.com/package/@ebisearch/baseline-portal) that contain the components needed to build new portals and can be customized via configuration files.

### ORCID claiming

ORCID [9] aims to be the main identifier for participants in research activities. Through it, research activities and outputs can unambiguously and persistently cite the contributors. In addition to the IDs themselves, ORCID maintains records of the contributions associated with each of its IDs. These records are normally updated manually by their owner by using the ORCID web page, a process called ‘ORCID claiming’ of research artefacts by their contributors. This process can be automated by using the ORCID REST API to facilitate the research pipeline.

Within EMBL-EBI, multiple resources implement ORCID claiming to allow contributors to associate data in EMBL-EBI resources with their ORCID ID. This is particularly interesting for data repositories where users deposit data to be referenced in publications. However, this causes each resource to implement its own integration with the ORCID APIs, resulting in development time being used by multiple resources to implement basically the same feature.

Since EBI Search already collects and stores information from multiple sources within EMBL-EBI to power its search engine, it is also capable of claiming this information to ORCID in a unified manner, thus allowing users of these resources to claim their data to their ORCID profile without the need for the resources to integrate with ORCID themselves. Users simply have to use the EBI Search page to find the entry that they want to claim and use the claim button to have this information in their profile. Claimed data are also shown for individual search results to link to their contributors. The Pathogens and COVID-19 Data Portals already use this feature to allow for claiming on search results.

## Updates to data resources

EBI Search provides metadata search functionality over EMBL-EBI’s data resources, as well as some data from outside EMBL-EBI. Data resources are arranged as a hierarchy of categories, each containing multiple domains (see the list in Supplementary Table S1). Since the previous update, AlphaFold [10], CancerModels.org [11], Infectious Diseases Toolkit (https://www.infectious-diseases-toolkit.org/), BioImage Archive [12], GEO DataSets [13], and the BioStudies repository [14] have been added as new resources. There have been a number of new categories created for general search: Images; Cohorts; Related resources; and Other data. Additionally, there are 13 new domains representing resources for the COVID-19 Data Portal and Pathogens Portal sites, many of them from outside EMBL-EBI. Some data resources have been deprecated, including HMMER [15]. A number of COVID-19-specific domains have also been retired, where content has been merged into the main dataset.

EBI Search retrieves updated datasets every 24 h, supplied in XML or JSON that follow defined schemas, or other formats. The data processing pipeline rebuilds most resources in under 6 h, ensuring that the results are timely.

The Summary (S4) service has been extended to include the AlphaFold dataset, allowing users to identify related entries from protein, protein structure, expression, and literature databases based on cross-references for AlphaFold entries. This allows users to find the related entries from other databases for the specific AlphaFold entry searched. In addition to identification, EBI Search also shows Summary metadata information of specific AlphaFold entries like Predictions, Model Structure, and Entities details, and provides the AlphaFold details page link where users can see more details about specific AlphaFold entries. The same information can be fetched using Summary APIs for further integration and investigation.

Additionally, EBI Search allows users to select AlphaFold entries and perform sequence similarity search using bioinformatics tools like BLAST and Dbfetch provided by the Job Dispatcher service. The seamless integration allows users to launch sequence similarity search for AlphaFold entries from the search result page.

## Discussion

Following the COVID-19 pandemic, EBI Search saw a surge in usage and an expansion in the data available to search: since the publication of the previous paper, the quantity of data indexed has grown from 5 billion to over 6.6 billion items. Maintaining the efficiency of the search engine while keeping abreast of technical updates is a challenge.

Very large datasets are also restricted by Lucene, which is limited to indexing 2.1 billion entries at a time. This has been worked around in the past by splitting the dataset into smaller subsections, but with increasing volumes of data an improved solution is necessary, and the team is investigating using an external search engine such as Solr (https://solr.apache.org/) to handle these datasets.

We expect that in the near future large language models and related technologies will provide opportunities to expand the search offerings available, further improving EBI Search flexibility and user experience.

## Supplementary Material

gkaf359_Supplemental_File

## Data Availability

EBI Search is available from https://www.ebi.ac.uk/ebisearch, while the API can be used interactively at https://www.ebi.ac.uk/ebisearch/documentation/rest-api.
