# Supplementary material for: EBI Search: providing discovery tools for biological metadata in 2025
Source: Nucleic Acids Res. 2025 May 5;53(W1):W273–6. doi: 10.1093/nar/gkaf359 (PMC12230726; doi:10.1093/nar/gkaf359)

Supplementary Table 1 - Data resources available in EBI Search in 2025

**Category**	**Data**
Genomes and metagenomes	DGVa, EGA, HGNC, LRG, MGnify, WormBase ParaSite, Ensembl, Ensembl Genomes
Nucleotide sequences	ENA, IMGT/HLA, NRNL1, NRNL2, IPD-KIR, IPD-NHKIR, IPD-MHC, GWAS Catalog, Rfam, RNAcentral
Protein sequences	UniProtKB, UniParc, UniRef, NRPL1, NRPL2, EPO, JPO, KIPO, USPTO
Macromolecular structures	AlphaFold, EMDB, PDBe, PDBe-KB
Bioactive molecules	ChEBI, ChEMBL, Ligands
Gene expression	Expression Atlases, ArrayExpress, dbGaP, GEO*, GEO DataSets*
Diseases	Human Diseases, OMIM, VarSite
Molecular interactions	Complex Portal, IntAct
Gene-disease associations	Open Targets
Reactions & pathways	BioModels, Cell Collective, MetaboLights, MetabolomeExpress, Metabolomics Workbench, Physiome Model Repository, Reactome, Rhea
Protein families	GPCRDB, Interpro, Pfam, TreeFam, MEROPS
Protein expression data	Cellosaurus, EVA, GNPS, GPMdb, iProX, jPOST, LINCS, MassIVE, NODE, PanoramaPublic, Paxdb, PeptideAtlas, PRIDE
Enzymes	Enzyme Portal, IntEnz
Literature	Europe PMC, Patent Families
Samples & ontologies	BioSamples, EFO, GO, MESH, OLS, Taxonomy
Catalogues & registries	Bio.tools, FAIRDOMHub, Identifiers.org registry, ORCID data claims
Genome variation	EVA Studies
Cohorts	CancerModels.org
Related resources	Infectious Diseases Toolkit*
Images	EMPIAR, BioImage Archive
Other data	BioStudies
* Data external to EMBL-EBI	

Supplementary Figure 1a – Top-level data coverage display showing categories and entry counts.

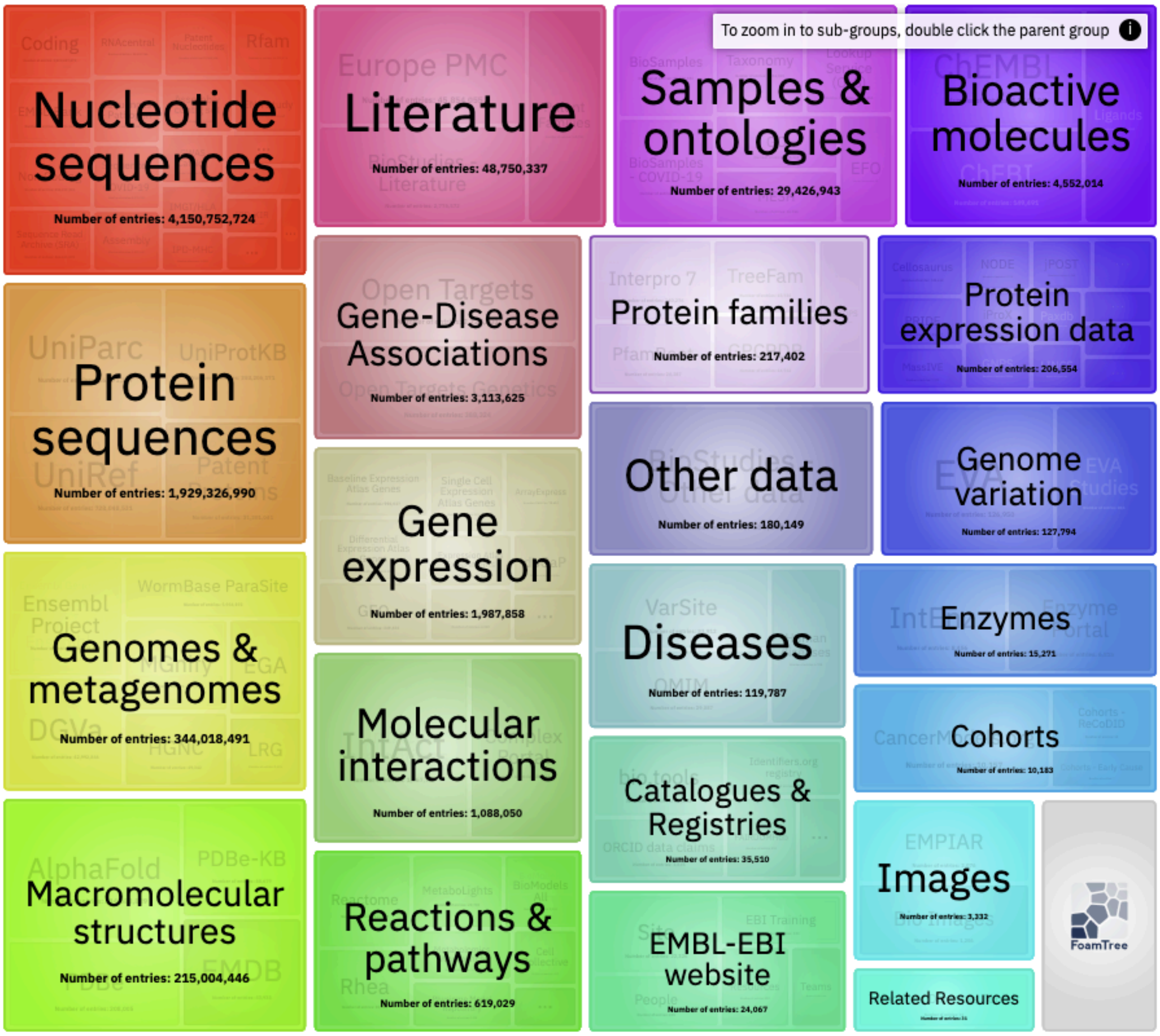

Supplementary Figure 1b – Data coverage display for Literature category.

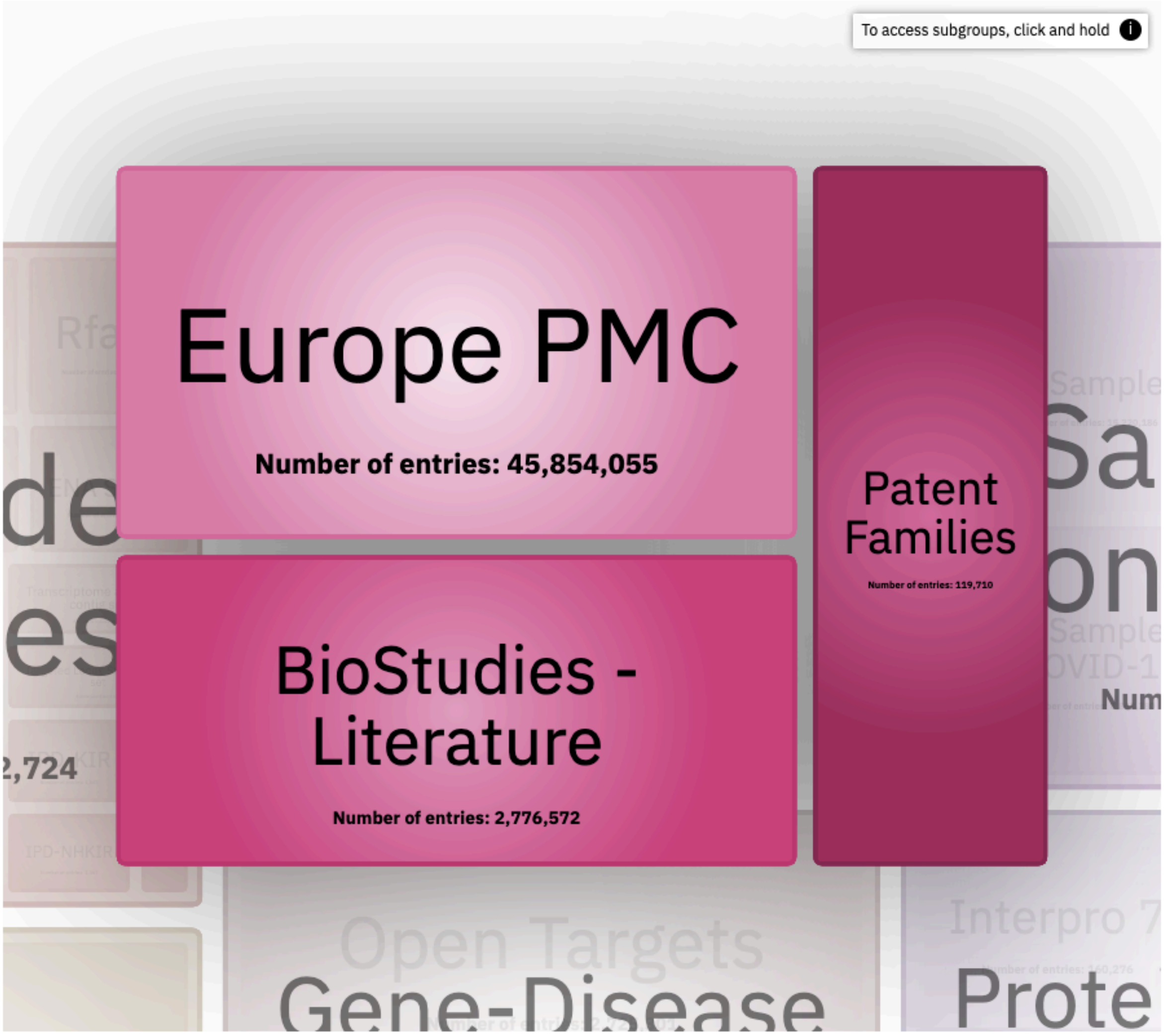

Supplement: gkaf359_Supplemental_File [file gkaf359_supplemental_file.pdf]
